# Gaussian-process-based Bayesian optimization for neurostimulation interventions in rats

**DOI:** 10.1016/j.xpro.2024.102885

**Published:** 2024-02-14

**Authors:** Léo Choinière, Rose Guay-Hottin, Rémi Picard, Guillaume Lajoie, Marco Bonizzato, Numa Dancause

**Affiliations:** 1 Department of Neurosciences and Centre Interdisciplinaire de Recherche sur le Cerveau et l’Apprentissage (CIRCA), Université de Montréal, Montreal, QC H3T 1J4, Canada; 2 Department of Electrical Engineering and Institute of Biomedical Engineering, Polytechnique Montréal, Montreal, QC H3T 1J4, Canada; 3 Mila - Québec AI Institute, Montreal, QC H2S 3H1, Canada; 4 Mathematics and Statistics Department, Université de Montréal, Montreal, QC H3T 1J4, Canada

**Keywords:** Neuroscience, Systems biology, Computer sciences

## Abstract

Effective neural stimulation requires adequate parametrization. Gaussian-process (GP)-based Bayesian optimization (BO) offers a framework to discover optimal stimulation parameters in real time. Here, we first provide a general protocol to deploy this framework in neurostimulation interventions and follow by exemplifying its use in detail. Specifically, we describe the steps to implant rats with multi-channel electrode arrays in the hindlimb motor cortex. We then detail how to utilize the GP-BO algorithm to maximize evoked target movements, measured as electromyographic responses.

For complete details on the use and execution of this protocol, please refer to Bonizzato and colleagues (2023).^1^

## Highlights

• A primer on Gaussian-process-based Bayesian optimization algorithm is presented
• General steps for online optimization of neurostimulation interventions are described
• An example intervention where an array and EMG wires are implanted in rats is provided
• Steps for online optimization of this specific intervention in a rat are described

## Abstract (editor highlights)

Publisher’s note: Undertaking any experimental protocol requires adherence to local institutional guidelines for laboratory safety and ethics.

## Graphical abstract

**Figure:**
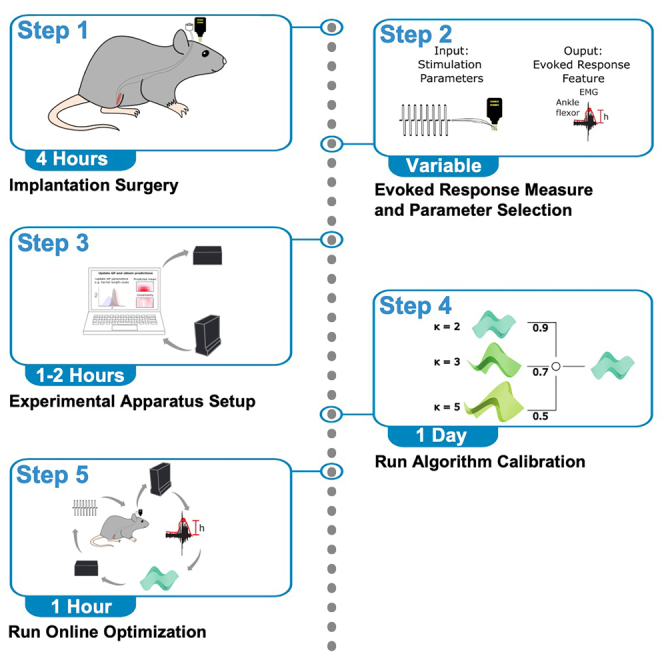

## Before you begin

### Background of the protocol

As demonstrated in the primary publication^1^ and elsewhere,^2^^,^^3^^,^^4^^,^^5^^,^^6^ Gaussian-process (GP)-based Bayesian optimization (BO) algorithms are a powerful framework to automatically optimize the efficacy of neurostimulation. It has been shown to outperform other strategies to simultaneously find the optimal values of multiple stimulation parameters (i.e., the optimal combination of parameter values) to maximize a chosen feature of the evoked response (e.g., the movement amplitude or the electromyographic [EMG] burst amplitude). The stimulation parameters can be of various nature, such as the electrode or electrical contact used to deliver the stimulation (i.e., stimulation location), the current amplitude, the pulse frequency, the pulse width or the timing of stimulation delivery during behavior.

In essence, stimulation optimization consists of the identification and selection of the stimulation parameter(s) value(s) with the greatest effect (efficacy) on the chosen feature of the evoked response. As the parameters-response relationship is treated as a black box, data points from the relationship are required to realize the optimization. Consequently, the complexity of the optimization process is determined by the number of parameters simultaneously explored and the number of potential values considered for each of these parameters (i.e., combinations of parameters values). Clinically, machine learning approaches hold great promise for applications that need solving of high-dimensional optimization problems, difficult or impossible to manually solve. However, to evaluate the performance of the algorithm and demonstrate its potential in a given application, simple scenarios in which only one parameter with few possible values is optimized, are desirable. In these cases, the parameter values can be systematically explored and the *ground truth* of the parameters-response relationship established. As an example, in the primary publication,^1^ after implanting rats with 32 electrodes multi-channel electrode arrays (MEAs) in the hindlimb motor cortex, we used the GP-BO algorithm to optimize the stimulation location (i.e., finding the best electrode) to maximize the EMG response amplitude recorded in a hindlimb muscle. In this scenario, we can easily stimulate each electrode and characterize the response to establish a *ground-truth* and quantify the performance of the GP-BO in comparison to other benchmark approaches. A similar research problem will be used below.

In the primary publication,^1^ we demonstrated that, in comparison to other benchmark approaches, the GP-BO converges to an adequate combination of parameter values using only a limited amount of data. This makes the framework particularly relevant to solve stimulation optimization problems when the total number of combinations of parameters values under consideration is large in comparison to the available number of stimulation queries. Moreover, the framework can be deployed online, in real-time, and is highly flexible. It can be applied to diverse optimization problems, without the need for extensive data collection beforehand. Accordingly, this approach can be very useful when electrodes are arbitrarily implanted (e.g., after a new MEA implant), or when the stimulation-response relationship evolves with time (e.g., as the disease progress such as in Parkinson’s or the brain reorganizes such as after stroke).

### Rationale of the protocol

As the GP-BO algorithm is a highly flexible algorithmic framework, it can be adapted for optimization in various neurostimulation paradigms with only minor changes to the methodology. To assist the reader in applying the framework to their specific paradigm, an overview of the general procedural steps is provided, followed by a comprehensive protocol detailing the specific procedures employed at each of these outlined steps. In particular, the protocol illustrates the optimization of parameters for intracortical microstimulation (ICMS) delivered using a MEA implanted in the hindlimb motor cortex in rats. The GP-BO algorithm optimizes the choice of electrode (i.e., stimulation location) to evoke a maximal amplitude of EMG response in a selected muscle.

To conduct our comprehensive protocol, you need to prepare the following equipment.

1. Materials for rat implantation procedure (MEA and EMG wires).
2. A surgery suite for the implantation procedure.
3. Tucker-Davis Technologies (TDT) electrophysiology apparatus (BioAmp processor, EMG amplifier and stimulus isolator) and a compatible Windows computer.
4. A dedicated space for electrophysiologic experimentation.

### Institutional permissions

All rat experiments follow the guidelines of the Canadian Council on Animal Care and are approved by the *Comité de déontologie de l’expérimentation sur les animaux* (CDEA, animal ethics committee) at Université de Montréal. Rats are housed in solid-bottom cages with bedding, nesting material, tubes and chewing toys. They are kept in groups of 3 before implantation and then individually thereafter to prevent housing partners from damaging the electrode array connectors during social grooming. They have ad libitum access to food and water and are subject to a 12:12-h light-dark cycle.

### Outline of the general steps

#### Theoretical background of the GP-BO algorithm

**Timing: depends on the experimenter | Hours to days**

In this step, familiarize yourself with the theoretical background of the GP-BO algorithm.

**Figure 1:**
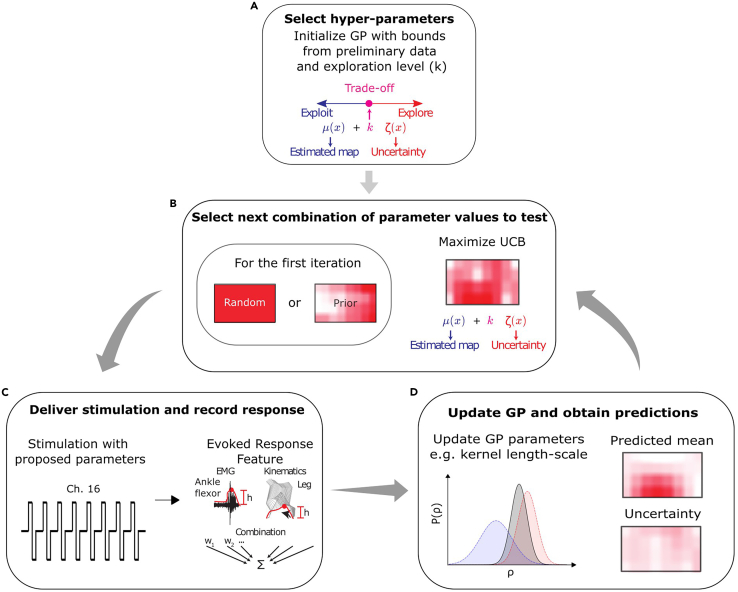
GP-BO algorithm procedure Steps involved in a GP-BO algorithm procedure are (A) the initialization of the GP with the selected hyperparameter values, corresponding to bounds on internal parameters and the value of the trade-off hyperparameter κ. (B) Next, the first combination of parameter values is selected randomly or based on prior knowledge. At every subsequent iteration, the combination to test is the one that maximizes the UCB. (C) Once the next combination to use has been determined, the stimulation is delivered accordingly, and the evoked response is recorded. (D) The parameters-response pairs are appended to the dataset which is fed to the GP. The predicted mean and uncertainty maps are updated as well as the internal model parameters by maximum likelihood estimation. The updated predicted mean and uncertainty maps are passed to the acquisition function, the UCB, to determine the next combination to test, and the loop goes on for a given number of queries.
  e. After the first stimulation query is performed (Figure 1C), the tested combination of parameter values and the recorded response are provided to the GP model, which integrates this new data and updates its internal parameters.
  f. Then, the model outputs a prediction of the response mean and the level of uncertainty for each combination (Figure 1D).
  g. Next, an acquisition function is used to select the next combination to query. This function assigns a score to each combination and selects the one associated with the optimal score to query.
    i. Here, as in in the primary publication,^1^ the Upper Confidence Bound (UCB) acquisition function is used. It is obtained by computing a weighted sum of the predicted mean and uncertainty.
    ii. The next combination to query is the one associated with the highest sum (Figure 1B, Right).
  h. This iterative procedure (i.e., query selection, stimulation delivery and model update) continues until the search is ended by the user, or a maximum number of queries are reached.
  i. The model is evaluated using two metrics computed after each query: the exploration performance and the exploitation performance.
    i. The exploration performance reports the efficacy of the combination of parameter values considered optimal by the algorithm (highest predicted response mean) in proportion to the optimum. When *ground truth* data is available, the efficacy of the optimal combination is known and can be compared to the efficacy of the combination predicted as the optimal by the model. In absence of *ground truth* data (e.g., online), the exploration performance is simply the efficacy of the combination predicted as optimal by the model.
    ii. ‘‘Exploitation’’ is the capability of targeting effective regions of the input space early and persistently. It follows that the exploitation performance metric reports the efficacy of the parameter choice taken by the algorithm at a given query in proportion to the optimum. When *ground truth* data is available, the efficacy of the queried combination is compared to the efficacy of the *ground truth* optimal combination. In absence of *ground truth* data, the efficacy of the queried combination is compared to the efficacy of the combination predicted as the optimal by the model.
  j. In the primary publication,^1^ the choice of kernel function, likelihood and acquisition function was tailored to the specific characteristics of the optimization task.

***Note:*** Alternative choices might yield better results in different scenarios. For example, if the user knows that the noise is not Gaussian, opting for a different type of likelihood could be more effective.

1. **GP-BO algorithm procedure.** In an iterative search procedure, the GP-BO algorithm models the relationship between combinations of stimulation parameter values (the input space) and their evoked responses (the scalar output space) using a GP.
  a. This model assumes that the responses follow a joint Gaussian distribution, where each combination is considered as a dimension of the multivariate Gaussian.
  b. A kernel function is used to define the covariance matrix of the multivariate Gaussian.
    i. This kernel function specifies how the responses to neighboring stimulation parameters values in the parameter space covary in the model.
    ii. For example, when a response has been obtained in an electrode, the kernel function determines to which extent the responses to neighboring electrodes in the MEA are predicted as similar in the model.
    iii. A popular kernel choice is Matérn’s,^7^^,^^8^ which was also used in the main publication.^1^
  c. Another important component of GPs is the likelihood function which models the noise in the data; a Gaussian likelihood was used in the primary publication.^1^
  d. Before beginning the iterative search, the GP is initialized (Figure 1A).
    i. When no prior information is available to inform the search, the mean of the GP is initialized as a uniform zero-valued function and the first combination of parameter values (i.e., the first “query”) is randomly selected across all possible combinations.
    ii. When a prior on the potential efficacy of combinations is available (e.g., a certain region of the electrode array is more likely to evoke a given movement), the GP-BO can be provided with this knowledge.
    iii. In the primary publication,^1^ the prior was constructed from responses obtained either in other subjects or in the same subject during previous optimization session(s). The construction comprised a map of expected response values for each combination. This map replaced the conventional uniform zero-valued mean function in the GP. In this case, the initial combination tested was not chosen randomly but based on the highest expected response from the map, as illustrated in Figure 1B (Left).

2. **GP-BO algorithm hyperparameters.** The search behavior of the algorithm is controlled by a set of hyperparameters that must be defined before running the algorithm.
  a. A first consequential hyperparameter is κ (Figures 1A and 1B), which controls the exploration-exploitation trade-off by differentially weighting the predicted mean and uncertainty in the UCB.
    i. A small κ biases GP-BO towards exploitation (i.e., repeated selection of known efficacious parameters). This could be chosen in situations in which the user prefers minimizing search time and increase treatment time. Combinations of parameter values that have been found to evoke good though perhaps suboptimal responses early in the search are favored.
    ii. A large κ biases GP-BO towards spending more queries exploring the input space. Combinations that have uncertain responses but are perhaps closer to the optimum are favored.
  b. The Matérn kernel is parametrized by ⍴, also referred to as the length-scale, which controls the propagation of information between neighboring combinations.
    i. A small length-scale implies low correlations; information propagation between neighboring combinations will be limited.
    ii. Inversely, a large length-scale implies high correlations; information propagation between neighboring combinations will make the model smoother.
  c. Furthermore, the noise of the Gaussian likelihood is parametrized by σ which controls the level of noise expected in the responses.
  d. The kernel length-scale ⍴ and the Gaussian likelihood noise σ can vary within predefined ranges. The lower and upper bounds of these ranges are defined as hyperparameters.
    ***Note:*** In the primary publication,^1^ we found that the performance of the algorithm was less sensitive to these hyperparameter values as compared to κ. However, they could have a more significant impact in other optimization paradigms.
  e. Finally, the number of combinations to test randomly at the beginning of the search is also a hyperparameter.

#### Animal or subject

**Timing: depends on the experimental setup | Hours to days**

In this step, prepare your animal or subject for the neurostimulation intervention. Specifically, this entails preparing the stimulation capabilities in the animal or the subject as well as ability to record and measure the evoked response feature.

Of course, the implementation of these capabilities will vary depending on the characteristics of your unique experimental paradigm. Some considerations should be factored in.

3. Determine what form the stimulation takes in the animal or subject. Does the animal or subject need to be implanted invasively or is the stimulation performed in a non-invasive manner? How is the stimulation parametrized? Can some parameters be fixed to known standard values?
4. Determine what are the effects expected to be evoked by the stimulation. How can this effect be measured? Does the subject or animal need to be implanted to measure and record this effect or can it be done in a non-invasive way?

#### Evoked response feature and optimized parameters

**Timing: depends on experimenter, however, might emerge naturally from a mature experimental setup**

In this step, identify the evoked response feature you want to maximize and the neurostimulation parameters that will be optimized for that purpose.

**Figure 2:**
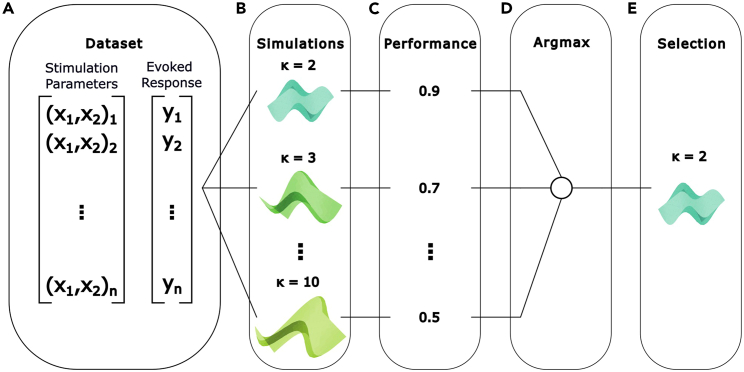
Hyperparameter calibration procedure Steps involved in the hyperparameter calibration procedure are (A) the acquisition of a valid dataset (previously collected data, semi-synthetic or synthetic). (B) The dataset is used to simulate the GP-BO algorithm search procedure using different values of the hyperparameter of interest, each for a given number of repetitions (10 or 50 is typically used). Other hyperparameters are fixed to default values determined as best in previous calibration procedures. (C) Once the simulations completed, the average performance at the final query is computed across repetitions for models associated with each hyperparameter value. (D) The performances associated with each hyperparameter values are compared. (E) The hyperparameter value associated with the highest performance is selected to be used in the online optimization procedure.

**Figure 3:**
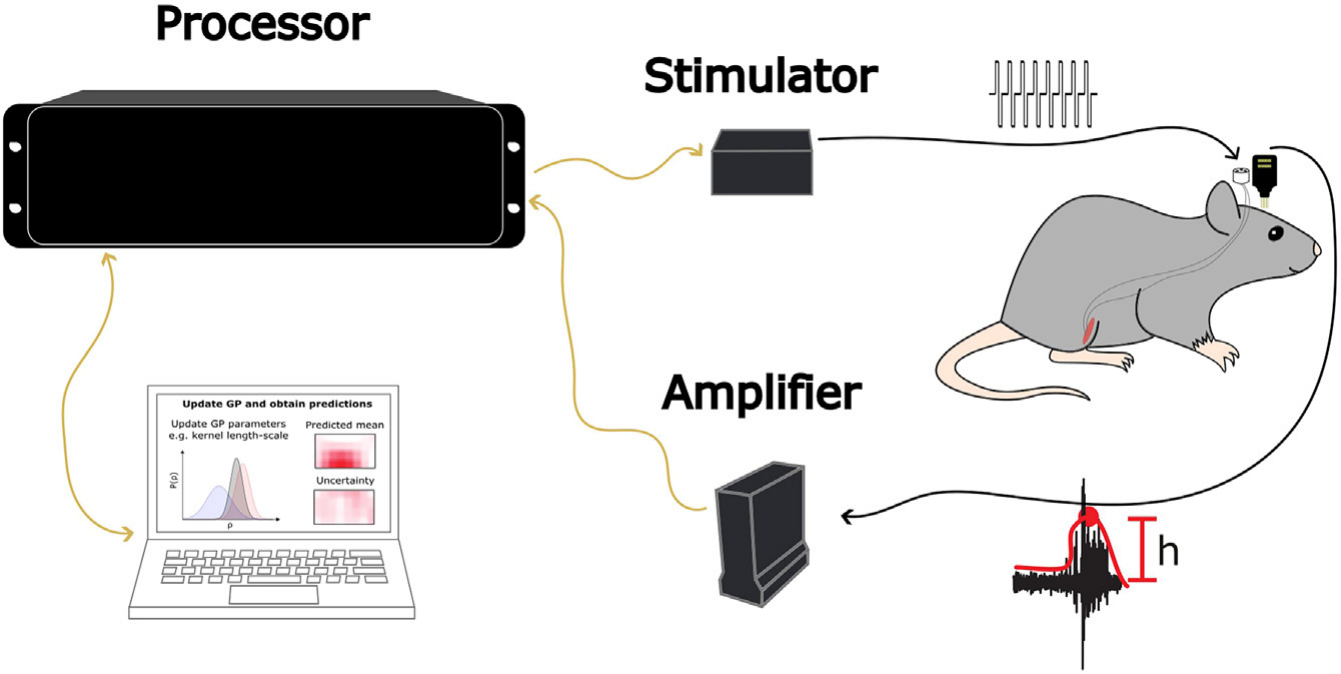
Tucker-Davis Technologies experimental apparatus with an implanted animal To realize the optimization procedure, the computer through the RZ2 BioAmp processor, provides stimulation parameters to the IZ2 stimulator and triggers the stimulation. The PZ5 EMG amplifier is then able to record the biomarker response and make it available to the computer through the RZ2. Since both the IZ2 and PZ5 share the same clock (the RZ2 clock), the response is easily aligned on the stimulation.
    iii. Whereas for a 5D spatiotemporal space that explored smoother parameters (i.e., pulse-width, frequency, train duration), 1% was enough (Fig. 8).
    iv. These results were obtained with a noise standard deviation corresponding on average to 25% of the maximum response (Fig. S3).
      ***Note:*** Using a procedure similar to hyperparameter calibration, the user can perform offline tests to estimate the required number of queries to reach a desired level of performance.
  d. Total number of combinations of parameter values in the parameter space:
    i. If only one parameter is optimized, the total number of combinations of parameter values is simply equal to the number of possible parameter values. As demonstrated in Fig. S8 of the primary publication,^1^ the total number of combinations of parameter values of the parameter space will influence the computation time, along with the number of queries required for the algorithm to converge to an optimal combination (solution).
    ii. Moreover, as the search progresses and more data are provided to the GP, the computation time will increase. As a reference, Figure S8C in the primary publication^1^ shows the relationship between increasing query history and computation time.
    iii. Overall, the total number of queries required depends on the desired optimization performance. The number of queries required to reach this performance will in turn depend on the total number of combinations of parameter values in the parameter space, the smoothness of the parameters-response relationship and the amount of noise in the responses.

5. Determine what is the evoked response feature of interest. The evoked response feature should quantify the stimulation effect and report its efficacy. The following factors should be considered when selecting the feature to measure:
  a. Validity: Keeping the ultimate goal of your neuroprosthetic intervention in mind, the feature should assess a quantity that closely matches the intended effect.
    ***Note:*** For example, if the intended effect of the stimulation is to make a limb muscle generate a movement, EMG activity in the muscle might act as a good proxy for the intended effect.
  b. Data type: The feature should be a scalar value, such as a floating-point number or an integer.
    ***Note:*** Although categorical features can be modeled within the GPBO framework,^9^ they require customization of the provided open-source library.
  c. Acquisition delay: Two factors determine the frequency at which the stimulation can be administered online.
    i. The first is the acquisition delay or how long after the stimulation can the feature be recorded.
    ii. The second is the processing delay associated with updating the model and obtaining the next combination of parameter values to query. This delay depends on the total number of combinations of parameter values and the computing equipment. As a reference, in the primary publication,^1^ we found that on a standard PC (e.g., Intel Core i7-9700K CPU@ 3.60 GHz), the 500th query of a 10k-combinations input space takes 130 ms. Using a GPU (e.g., NVIDIA ®GeForce ®RTX 2080), this computation time is reduced to 7 ms (see Fig. S8 in the primary publication^1^).
    iii. The acquisition delay will increase the optimization time and potentially limit the number of queries that can performed.
      ***Note:*** Since several queries might be required to converge to an optimal combination, prioritizing an evoked response feature that can be obtained within a short delay is advisable if the experiment is time constrained.
  d. Stability: The parameters-response relationship should be stable across minutes to hours to allow the convergence to a solution within an optimization session.
    ***Note:*** When the relationship is not stable over longer timescales, optimization sessions can be repeated to track the solution as it changes. See Fig. 5 in the primary publication^1^ for more details on this repeated optimization procedure.
6. In a well-posed optimization problem, the input parameters should have a marked impact on stimulation efficacy. Once the parameters to optimize have been selected, consider the following factors:
  a. Number of possible parameter values: The algorithm optimizes parameters within a discretized range of potential values. Define a plausible range and the level of granularity within this range.
  b. Parameter representation in the input space: A given parameter might vary within a discrete range. However, careful consideration must be used to find the best representation of the parameter within this range.
    ***Note:*** For example, if the stimulation location is the parameter of interest, representing each spatial dimension as an independent parameter is advisable. For an electrode in a 2D MEA, the first parameter would be the position along the x axis and the second the position along the y axis. This representation is richer since it allows propagation of information between neighboring stimulation locations and does not collapse two dimensions into a single range.
  c. Characteristics of the parameters-response relationship: The smoothness (the degree of similarity between responses to neighboring parameter values) of the parameters-response relationship and the noise level present in responses will also influence the number of queries required for convergence.
    i. In general, a smooth relationship with low noise allows faster convergence.
    ii. As a reference, we found that a number of queries corresponding to 25%–50% of the total number of combinations of parameter values was required when optimizing the 2D spatial location of the stimulation in the presence of a non-smooth relationship, which we argue is the worst-case scenario for our application in the primary publication^1^ (Figures 2 and 3 of the primary publication).

#### Experimental apparatus

**Timing: hours to a day depending on the apparatus**

In this step, configure the experimental apparatus to generate the adequately parametrized stimulation and record the evoked response feature.

7. Prepare a computer to run the algorithm and control the experiment.
  a. To run the algorithm, the computer requires the appropriate configuration of our published library (see key resources table).
  b. To control the experiment, the computer must interface with the electrophysiological components of the experimental apparatus.
8. Prepare an electrophysiological apparatus to generate the adequately parametrized stimulation once instructed by the computer.
  a. For low-latency-short-duration evoked response features, it is important that this system delivers the stimulation trigger to the system that records the response. This allows the appropriate alignment of the response on stimulation onset.
  b. This stimulation generating element of the apparatus must be addressable from within Python to parametrize the next stimulation and trigger it.
9. Prepare a system, possibly electrophysiological depending on the nature of the evoked response feature, to record the response and make it available to the library so the scalar feature can be extracted and passed to the algorithm.
  a. This response recording element of the apparatus must be addressable from within Python so the value of the feature can be extracted, passed to the algorithm and the loop can proceed.

#### Run algorithm calibration procedure

**Timing: 1 day**

In this step, prepare the algorithm for the optimization of your parameters of interest by simulating the procedure with preliminary data. The primary objective here is to calibrate the hyperparameters values or in other words find which values are likely to work well online. This is a crucial step for achieving optimal performance within a new experimental paradigm.

10. Determine the data source (Figure 2A). To run the hyperparameter calibration procedure, three main options are available:
  a. Pre-collected data: This implies using data for which the parameters to optimize are systematically varied while recording the evoked response feature.
    ***Note:*** At this stage, since the experimental apparatus has been configured in the previous section, collecting preliminary data is possible.
  b. Semi-synthetic data: This is possible when you have access to pre-collected though incomplete datasets that can be artificially augmented to complete them.
    i. For instance, consider a dataset in which each parameter value has been used to stimulate for a limited number of repetitions (e.g., n = 3).
    ii. First, the mean evoked response feature can be computed for each parameter value.
    iii. Next, parameterized noise can be added to the mean response features to simulate a dataset in which each parameter has been used to stimulate for a sufficiently large number of repetitions (e.g., n = 20).
  c. Fully synthetic data: This implies generating data based on your knowledge of the system.
    i. For each combination of parameter values considered, plausible evoked response features must be assigned. Care must be taken to replicate the properties of the parameters-response relationship, such as smoothness and noise levels.
    ii. This task can be challenging, as it involves relying on partial knowledge of the black-box system. In some cases, this information can be found in other publications. For example, Watson et al. provide insight on the effect of cortical stimulations on EMG responses.^10^^,^^11^^,^^12^
11. Prepare hyperparameter calibration. After acquiring the data, you can simulate the execution of the algorithm using different values of the hyperparameters and determine which values yield the best performance (Figures 2B–2E). Hyperparameters that are amenable to optimization are:
  a. κ:
    i. We found that the performance of the algorithm exhibited the most sensitivity to this hyperparameter (see Fig. S1 of the primary publication^1^).
  b. Bounds on the length-scale (⍴) and on the likelihood noise (σ):
    i. We found that the performance of the algorithm was less sensitive to these hyperparameters (see Fig. S1 of the primary publication^1^) however this may not be the case in new experimental paradigms.
    ii. Nevertheless, if prior knowledge about the smoothness of the parameters-response relationship exists, bounds for the length-scale can be biased toward higher or lower values to respectively reflect a smoother or coarser space.
    iii. Similarly, if a high or low level of noise is expected in the responses, bounds for the noise parameter of the Gaussian likelihood can be respectively biased toward larger or lower values.
  c. The number of queries to perform:
    i. As previously stated, the total number of queries depends on the desired optimization performance.
    ii. The number of queries required to reach this performance will in turn depend on the total number of combinations of parameter values, the smoothness of the parameters-response relationship and the amount of noise in the responses.
    iii. To estimate the performance of the algorithm after a certain number of queries, different values can be assessed in a procedure similar to hyperparameter optimization.
12. Run hyperparameter calibration.
  a. Verify the installation of our published library (see key resources table) with an appropriate Python virtual environment.
  b. The selection of hyperparameters to calibrate as well as where to load the data from is configured by populating a config file.
  c. Run the hyperparameter calibration command. Example commands can be found in **“EduOptimNeurostim/tutorials/Experiment1.ipynb”.**

#### Run online optimization procedure

**Timing: minutes to hours**

In this step, deploy the algorithmic framework to optimize the stimulation parameters in the animal or subject online.

13. Test the software-hardware apparatus.
  a. Is the stimulation adequately parametrized and triggered?
  b. Is the evoked response feature adequately recorded?
  c. Is the algorithm adequately updated with the new data?
14. Bring the animal or the subject to the experimentation space.
15. Connect the stimulation and the recording apparatus so it interfaces appropriately with the animal or the subject.

***Note:*** The setup should ensure a stable parameters-response relationship. For example, the animal might be sedated or habituated to remain calm while gently held.

16. Run the algorithm using the optimal hyperparameters found in the previous section.
17. Assess the evolution of the exploration and exploitation metrics across queries and the combination of parameter values found as optimal after the last query in the output plots.
18. For examples of the online optimization procedure using the GP-BO algorithm, see Video S1 and S2 in the primary publication.^1^

## Key resources table

**Table:** 

REAGENT or RESOURCE	SOURCE	IDENTIFIER
**Chemicals, peptides, and recombinant proteins**		

Baytril 100 (enrofloxacin 100 mg/mL)	Bayer	DIN: 02249243
Dexacort 5 (dexamethasone sodium phosphate 5 mg/mL)	Rafter 8 Products	DIN: 02314118
Isoflurane USP 250 mL	Fresenius Kabi	DIN: 02237518
Rheumocam (meloxicam 5 mg/mL)	Merck	DIN: 02413868
Vetergesic (buprenorphine hydrochloride 0.3 mg/mL)	Ceva	DIN: 02342510
Xylocaine (lidocaine hydrochloride 20 mg/mL)	Aspen Pharmacare Canada	DIN: 02302438
Proviodine solution (povidone-iodine 10% solution)	Teva	DIN: 00172944
Lactated ringer’s injection	B. Braun	DIN: 01931636
Optixcare eye lube	Aventix	Cat#5914304
Ortho-jet powder (fast curing orthodontic acrylic resin powder, 454 *g*)	Lang Dental Manufacturing Company	ID: 1330
Ortho-jet liquid (fast curing orthodontic acrylic resin liquid, 946 mL)	Lang Dental Manufacturing Company	ID: 1306

**Software and algorithms**		

Synapse∗	Tucker-Davis Technologies	https://www.tdt.com/support/downloads/
RPVDSex∗	Tucker-Davis Technologies	https://www.tdt.com/support/downloads/
Published library∗	Bonizzato et al.^1^	https://github.com/mbonizzato/EduOptimNeurostim

**Deposited data**		

Rat and NHPs cortical stimulation datasets∗	Bonizzato et al.^1^	https://osf.io/54vhx/

**Experimental models: Organisms/strains**		

Female or male Long-Evans rat (275–350 *g* at surgery)	Charles River Laboratories	Strain Code: 006

**Other**		

Ethilon nylon suture 4-0 (45 cm)	Ethicon	Product Code: 662SLH
Absorbable coated Vicryl suture 4-0 (45 cm)	Ethicon	Product Code: J386H
Screws	McMaster-Carr	Cat#96817A704
Gelfoam size 12 (12 cm^2^)	Pfizer	Manufacturer Code: 09-0891-01-005
Ultra-precise small animal stereotaxic instrument	Kopf Instruments	Model 963
Rat anesthesia mask (compatible with the stereotaxic instrument)	Kopf Instruments	Model 906
Isoflurane anesthesia machine	Dispomed	SKU: 975-0510-000 / 965-0500-000 /990-VI5K-SVEEK
Rodent mask	Dispomed	SKU: 980-0200-082 / 904-1050-010
Induction chamber	Dispomed	SKU: 904-1040-000
Dumont #5SF forceps	Fine Science Tools	Item No. 11252-00
Needle holder	Fine Science Tools	Item No. 12001-13
Small scissors	Fine Science Tools	Item No. 14184-09
Dura scissors	Fine Science Tools	Item No. 15002-08
Rongeur	Fine Science Tools	Item No. 16221-14
Slim elongated needle holder	Fine Science Tools	Item No. 12005-15
Homeothermic blanket	Harvard Apparatus	Item No. 55-7020
Germinator 500 glass bead sterilizer	CellPoint Scientific	Product No. 5-1450
Hand drill	Foredom	SKU: K.1070
Carbide burs HP-1/2	SS White	SKU: 14821
Screwdriver matching screws	Moody Tools	SKU: 51-2089
Wide-field surgical microscope	Zeiss	Model S100-OPMI pico
Small pet hair grooming trimmer	Oneisall	ASIN: B089W594LC
EMG wire assembly∗	Omnetics	DWG No: A118591-001
Omnetics connector∗	Omnetics	DWG No. A22005-001
Mini-DB26 connector∗	TE Connectivity	TE Internal #5749621-2
Isolated pulse stimulator	A-M Systems	MODEL 2100
ZIF-Clip based microwire arrays∗	Tucker-Davis Technologies	Part No. ZIF2010-32
32-channel digital ZIF-Clip headstage holder∗	Tucker-Davis Technologies	Part No. ZCD-ROD32
RZ2 BioAmp processor∗	Tucker-Davis Technologies	N/A
IZ2 Stimulator 32-channels (IZ2-32)∗	Tucker-Davis Technologies	N/A
PZ5 Neurodigitizer amplifier∗	Tucker-Davis Technologies	N/A
32-channel aluminum ZIF-Clip headstage∗	Tucker-Davis Technologies	Part No. ZC32
PO5E interface card∗	Tucker-Davis Technologies	N/A
Computer (Windows)∗	N/A	N/A

***Alternatives:*** Products marked with a ∗ in the table were used to perform the experiments in our laboratory and are recommended for the successful completion of the protocol. Other items without the symbol can be easily replaced with alternatives.

## Step-by-step method details

### Animal or subject | rat implantation surgery

**Timing: 4 h**

The purpose of this step is to implant a rat already habituated to human interaction with a MEA in the hindlimb motor cortex as well as subcutaneous EMG wires in a leg muscle. This is a model used to study neuroprosthetic interventions for rehabilitation after spinal cord injury.^13^ All the following surgical procedures must be conducted aseptically.

#### Prepare the animal

**Timing: 30 min**

The purpose of this sub-step is to induce anesthesia and prepare the animal for the EMG and MEA implantation.

1. Lay the homeothermic blanket under the stereotaxic frame and cover it with surgical drapes.
2. Weigh the rat.
3. Induce anesthesia using the induction chamber. Set the oxygen level to 1 L/m and increment the isoflurane level 1% every 30 s until the level reaches 5%.
4. Once the animal is well sedated, transition to the rodent mask on ∼2% isoflurane.

**CRITICAL:** For the rest of the surgery, adapt the isoflurane level as a function of the arousal of the animal. Assess the arousal by the presence of a pain reflex when pinching the hind paw. There should be no response. The isoflurane level should be as low as possible while maintaining adequate anesthesia.

5. Apply eye lube with sterile Q-tips to keep the eyes moisturized and protected from dust and hairs.
6. Use the hair trimmer to shave the fur off the hind limb of interest as well as off the head and the neck. Take precautions to not shave the whiskers or to damage the mammary papillae.
7. Apply proviodine to the skin of the shaved areas using sterile wipes.
8. Place the rat into the stereotaxic frame and connect the anesthesia machine to the stereotaxic frame mask.
9. Administer dexamethasone (1 mg/kg) intramuscular in the Gastrocnemius muscle of the leg opposite to the leg of interest to prevent inflammation.
10. Administer enrofloxacin (10 mg/kg) intramuscular in the Gastrocnemius muscle to prevent infections.
11. Inject saline (5 mL/kg/h) subcutaneously to hydrate the animal during the procedure.
12. Manage the animal’s temperature.
  a. Lubricate the temperature probe of the homeothermic blanket with petroleum jelly and insert it into the rectum of the rat.
  b. Secure the probe to the tail using surgical tape.
  c. Turn on the homeothermic blanket.
13. Monitor the vital signs of the animal frequently (every 10 min) for the remainder of the surgery. Temperature should be maintained around 36.5°C.

#### EMG implantation

**Timing: 1–2 h**

The purpose of this sub-step is to implant the Tibialis Anterior with a pair of subcutaneous EMG wires to record the muscle activity evoked by stimulation.

14. Immerse the EMG wire assembly and the TDT microwire array (MEA) in 70% isopropyl alcohol. Remove from the alcohol and thoroughly dry.
15. The surgical procedure must be conducted aseptically.

**CRITICAL:** Every surgical tool going in the animal should be sterilized beforehand or go through the bead sterilizer.

16. Dilute 20 mg/mL Lidocaine 1:4 with sterile water to get a final concentration of 5 mg/mL.
17. Prepare to cut the skin along the midline of the skull, from the imaginary line connecting the eyes to the back of the head. Inject 3 mg/kg of lidocaine subcutaneously (e.g., 0.2 mL of the 5 mg/mL solution for a 300 *g* rat).
18. Make the incision with a #15 blade mounted on the scalpel. If needed, elongate the opening with the small scissors. This incision must reveal the skull above the motor cortex where a craniotomy will later be performed.
19. Prepare to make a small incision in the skin of the hindlimb above the Tibialis Anterior muscle. Inject 3 mg/kg of lidocaine subcutaneously (e.g., 0.2 mL of the 5 mg/mL solution for a 300 *g* rat).
20. Make the incision using the #15 blade mounted on the scalpel again.
21. Use the slim elongated needle holder to subcutaneously thread a pair of EMG wires from the head incision to the incision above the Tibialis Anterior.
  a. Pull the pair of microwires out of the skin.
  b. Create subcutaneous space around the leg incision by carefully opening the connective tissue by opening the small scissors and using the blunt exterior of their blade.

***Note:*** This will allow the placement of a loop in the EMG wires after their implantation, preventing direct tension when the animal is moving.

22. Thread the wires through the muscle.
  a. With the needle holder, gently bend two 23G needles to form a rough arc.
  b. Insert the needles through the muscle, making sure they travel a parallel and adjacent trajectory through the muscle.
  c. Thread the wires into the bevelled tip of the needle and pass the wires through the muscle by pulling the needles back out.
23. Remove a small portion of insulation on the wires using the #15 blade approximately 0.5 cm before their extremity to create an electrical interface.
24. Secure the wires into the muscle.
  a. Tie their extremities together with a tight knot using the nylon 4‒0 suture thread.
  b. Pull the uninsulated section of the wires back into the muscle to make the knot sit close to the muscle.
  c. Again, using nylon 4‒0 suture thread, tie another tight knot around the wires where they entered the muscle. The EMG wire insulation can be slightly stretched manually to have it protrude < 0.5 mm the wires ending.

***Note:*** This will ensure the absence of any electrical contact at the wire tip.

25. Confirm the appropriate implantation of the muscle.

**CRITICAL:** Verify the electrical contact with the appropriate muscle by visualizing an adequate motor response to a brief train of electrical stimulation applied through the implanted wires. The movement response should be clearly visible and specific to the target muscle. For the Tibialis Anterior, expect a clear ankle dorsi-flexion. To apply the stimulation, connect the wires to the isolated pulse stimulator and send a stimulation parametrized as to do ICMS: a train of 13 cathodal 200 μs duration square pulses with an inter-pulse interval of 3.3 ms. To ensure the quality of implantation, movements should be evoked with stimulation intensities <300 μA.^14^

26. Gently insert the wire under the skin while making sure to leave a 1–2 cm loop in the wires to prevent direct tension.
27. Suture the incision skin with absorbable suture thread.
28. Insert 2 ground wires subcutaneously over the fatty tissue in the torso using the slim elongated needle holder.

#### Electrode implantation

**Timing: 1–2 h**

The purpose of this sub-step is to implant a MEA in the hindlimb representation of the motor cortex.

29. Coming back to the head incision made earlier, dissect away all periosteum from the skull using the small forceps and the small scissors and/or by scrubbing the skull with sterile Q-tips.
30. Outline a 2 (medio-lateral [ML]) × 3 (antero-posterior [AP]) mm window, contralateral to the implanted hindlimb, to drill above the region of interest of the motor cortex using a pencil.

**CRITICAL:** The most antero-medial corner of the rectangle window should be positioned at ‒1 mm AP and +1 mm ML to bregma.

31. Under the microscope, using the hand drill with a size HP-½ carbide burr adjusted so it protrudes only 2 mm from the drill head, drill four holes in the corner of the window.
32. Drill four additional holes for screw insertion. Two holes anterior to the window, each on opposing sides of the medial line of the skull. Idem for two holes posterior to the window.
33. Using the screwdriver, install the screws in the holes making sure they are stable however don’t go too deep (approximately 1.5 turns deep once the screw bites).
34. Complete the craniotomy. Using the rongeur, pinch between the holes to cut the bone.
  a. Position the tip of the jaw of the rongeur in adjacent holes.
  b. Apply gentle pressure and pull up away from the skull before applying sufficient force to close the rongeur, cutting the bone and connecting holes along an edge of the window.
  c. Repeat for the other three edges.
35. Use the small forceps to remove the remaining bone flaps and then the rongeur to clean the edges of the window. Be careful not to damage the surface of the brain. At this point, if the brain shows signs of edema, see **Troubleshooting problem 1** below.
36. Verify the placement of the MEA with regards to the window.

**CRITICAL:** In our study, the most antero-medial site of the MEA was positioned at coordinates AP ‒1.1 mm, ML +1.3 mm from bregma with its length in the AP axis, however hindlimb representation can also be found up to 1 mm anterior to these coordinates.^13^

37. Measure the origin or the zero of the dorso-ventral (DV) coordinate of the MEA placement.
  a. To do so, mount the MEA into its headstage holder on the stereotaxic manipulator and descend it until it contacts the exposed dura.
  b. Note the measurement and raise back the MEA.
38. Open the dura.
  a. Using the needle holder, gently bend the 30-gauge needle and use the needle to carefully initiate a cut in the dura.
  b. From the cut, remove the dura using the small forceps and the small Vannas scissors to fully expose the brain in the window.
39. Using the stereotaxic manipulator, lower the MEA back to the measured DV zero.
40. Twist the two ground wires of the MEA around 2 of the 4 screws.
41. Still using the stereotaxic manipulator, descend the MEA to a 1.5 mm depth in the brain.
42. Cover the exposed brain around the MEA using Gelfoam. Carefully hydrate the Gelfoam using sterile ringer saline before applying it onto the brain.
43. Use acrylic powder and liquid to build a small protective hat above the craniotomy making sure it is properly anchored to the four screws and encases the MEA and EMG connector.

***Note:*** The acrylic hat should be as smooth as possible and cover the skull to fill the opening made by the incision. Wait for the acrylic to harden.

44. Gradually decrease the isoflurane anesthesia and remove the animal from the stereotaxic frame while continuously monitoring vital signs and ensuring the body temperature of the animal remains stable while the animal is waking up.
45. Administer meloxicam (1 mg/kg) subcutaneously. Administer buprenorphine (0.025 mg/kg) subcutaneously.
46. Continue with a daily dose of meloxicam (1 mg/kg) and enrofloxacin (10 mg/kg) for 3–4 days post-surgery on an appropriate schedule.

### Evoked response feature and optimized parameters | EMG envelope peak amplitude and ICMS electrode location

**Timing: depends on the experimenter**

In this step, the EMG envelope peak amplitude is selected as the evoked response feature and the stimulation electrode location as the ICMS parameter to be optimized.

47. The EMG activity feature reports stimulation efficacy. The following factors are taken into consideration:
  a. Validity: EMG activity serves in this context as an adequate proxy for targeted muscle contraction, which is the actual target to maximize.
  b. Data type: Even if the EMG activity is a complex signal, its envelope can be computed, and a simple feature of the envelope can be obtained. Specifically, the peak amplitude of the envelope evoked by the stimulation is used as the scalar response.
  c. Acquisition delay: Since the EMG activity decays rapidly after the offset of the stimulation, the amplitude of the envelope can be computed and recorded in less than a second.
  d. Stability: Given a successful implantation surgery, responses to stimulation can be obtained across multiple days to weeks. Even if EMG responses are noisy, the parameters-response relationship is sufficiently stable for the algorithm to effectively operate in a given session.

***Note:*** The solution might evolve over time, for example as expected after an injury, and additional runs of the algorithm might be required to track these changes. In these cases, instead of initiating the search *tabula rasa*, previous data can be used as prior to accelerate the search (see Fig. 5 of the main publication^1^ ).

48. The stimulation location parameter in the MEA is the electrode contact from which the stimulation is delivered. The following factors are taken into consideration:
  a. Number of possible parameter values: Here the use of a 32-electrode MEA constrains the number of possible parameter values to 32 as each electrode only has one contact.
  b. Parameter representation in the input space: To respect the topographical arrangement of the electrodes, each spatial dimension is taken as an independent parameter. It follows that the first and second parameters are the vertical and horizontal coordinates of the electrode respectively (the TDT MEAs used have 8 rows by 4 columns of electrodes).
  c. Characteristics of the parameters-response relationship:
    i. Because of the mosaic nature of movement representation in the motor cortex,^15^^,^^16^^,^^17^^,^^18^^,^^19^ the relationship between stimulation electrode location and the EMG responses can be quite unsmooth.
    ii. Specifically, this phenomenon is illustrated by neighboring electrodes evoking very different movements (e.g., ankle dorsiflexion versus hip external rotation), and consequently EMG responses in the Tibialis Anterior muscle (respectively resulting in a strong versus weak response) (see Fig. 2B of the primary publication^1^).
    iii. In contrast, stimulation frequency or stimulation duration results in smoother parameters-response relationship (see Fig. 7D of the primary publication,^1^ right panel).
  d. Total number of combinations of parameter values in the parameter space: Even if the single parameter to optimize is represented using two dimensions, the total number of combinations of parameter values in the parameter space is still 32 (8 ×4).

### Experimental apparatus | Tucker-Davis Technologies

**Timing: 1–2 h**

The purpose of this step is to prepare the experimental apparatus to emit the stimulation parametrized adequately when triggered and record the evoked response feature.

49. In the space for electrophysiologic experimentation, prepare the Windows computer.
  a. Install the PO5E Interface Card to interface with the TDT hardware.
  b. Update Windows.
  c. Ideally, plug in two monitors.
50. Interface the computer with the electrophysiology apparatus following the wiring schematic in Figure 3.
  a. Plug in the RZ2 BioAmp Processor in the computer using an optical fiber cable.
  b. Plug in the IZ2 Stimulator in the RZ2 using an optical fiber cable. Plug in the 32-channel ZIF-Clip headstage into the IZ2.
  c. Plug in the PZ5 Neurodigitizer Amplifier in the RZ2 using an optical fiber cable.
  d. To interface the implanted EMG wire assembly with the PZ5, a custom headstage is required.
    i. The headstage is composed of two connectors; an Omnetics connector on the animal and a Mini-DB26 on the PZ5 (See key resources table).
    ii. Wire the connectors together using the following instructions and plug the resulting headstage into the PZ5: https://www.tdt.com/docs/technotes/tn/TN0896/.
51. Install the required software.
  a. Turn on the RZ2, the PZ5 and the IZ2.
    ***Note:*** When booting the PZ5, set the mode to EMG and the acquisition frequency to 6 kHz. Synapse expects to receive a signal at this frequency during the algorithmic online procedure.
  b. Install the TDT Drivers/RPvdsEx and Synapse (See key resources table).
  c. Install our GitHub library into an appropriate folder (See key resources table).
52. Configure Synapse.
  a. Open Synapse.
  b. Detect the available hardware.
    i. Click “Menu >” > “Edit Rig”. Click “Detect”.
    ii. The detected hardware is displayed in a tree containing the PC at the root, the RZ2 down a branch and its associated PZ5 and processors.
  c. Add the IZ2.
    i. By default, Synapse does not detect the IZ2.
    ii. Click on one of the 8 processors (e.g., “**DSP8”**) and change the processor model to “**DSPI”** using the dropdown menu.
    iii. Right click on the newly configured “**DSPI”** (e.g., “**DSPI8”**) processor and click on “**Add IZn**”.
    iv. Verify the “**Model**” and “**Channels”** of the IZ2 have been appropriately configured.
    v. Click “**OK**” to exit the rig editor.
  d. Load the provided circuit in Synapse.
    i. Click on the “**Experiment**” button (the logo right under “**Menu >**” at the top left) > **“Import Experiment”.** Select the file at “**EduOptimNeurostim/Synapse/circuit.synexpz**”.
    ii. This circuit implements control of the stimulation parameters using the **“eStim1”** gizmo.
    iii. The EMG signal is streamed by the “**NPro1**“ gizmo from the PZ5, a specific muscle signal is selected using the **“Sel1”** selector gizmo.
    iv. This signal is sent to the “**buffer_1**” gizmo which captures the peri-stimulus signal and enables its retrieval.
    v. Synapse provides a Python API enabling the interaction with variables of the gizmo.
53. Verify the configuration of the circuit.
  a. Are the default values for stimulation parameters appropriate?
    i. Click on the **“eStim1”** gizmo and open the “**Stim Voices**” tab. Verify the parameterization of the stimulation.
    ii. By default, the circuit sets the pulse duration at 200 μs, the interpulse period at 3 ms, the pulse amplitude at 30 μA and the number of stimulation pulses in the train burst at 13.
  b. By default, the circuit saves both the EMG signal using the “**NPro1**'' gizmo and the stimulation waveforms using the “**eStim1**” gizmo in a data tank. The acquisition frequency of these saved signals is controlled at different levels.
    i. Clicking on the RZ2 icon in the **“Processing Tree”** and clicking on the **“Main”** tab, you can adjust the “**Master Device Rate**" which will set an upper bound for the IZ2 and PZ5 specific rates.
    ii. Then, you can adjust the sampling rate for the EMG signal in the “**NPro1**'' gizmo **“Storage”** tab.
    iii. Similarly, you can adjust the sampling rate for the stimulation waveform in the “**eStim1**” gizmo **“Misc and Saving”** tab.
54. For any problem relating to Synapse, see **Troubleshooting problem 2** below.

### Run algorithm calibration procedure | calibration of the trade-off hyperparameter κ

**Timing: 1 day**

The purpose of this step is to find the value of κ that is most likely to work best when optimizing the electrode location used to evoke maximal peak EMG amplitude in the Tibialis Anterior online. Using pre-collected data in which the 32 electrodes have been stimulated multiple times and the EMG responses have been recorded, the algorithmic procedure using different values of κ can be simulated and the value which works best can be identified.

55. Install the library and navigate to it using the Command Prompt.

> cd <Appropriate_Folder>

> git clonehttps://github.com/mbonizzato/EduOptimNeurostim.git

> cd /EduOptimNeurostim

56. Follow the instructions in the “**README”** of the library to install the appropriate version of Python and the dependency libraries using virtualenv. Alternatively, the virtual environment can be created using Anaconda.

> virtualenv --python=python3.7.4 <PATH_TO_ENVIRONMENT>

> <PATH_TO_ENVIRONEMENT>/venv/Scripts/activate

> pip install -r requirements.txt

57. From the **“/config”** folder, create your own config file by copying “/**online.json”** to create “/**config.json”**.
58. The following steps imply calling different versions of the algorithm. The calls are always made through the **“/main.py”** script and the behavior of the script is controlled by the **“/config.json”** file. This file contains key-value pairs that specify various aspects of the behavior of the algorithm. The essential ones are listed below, with a complete list available in the **“README”**.
  a. To control where the outputs of the algorithm are saved in the **“/EduOptimNeurostim”** directory, the value associated with the key **“output_path”** is used.
    "output_path": "output/rat_mapping_2D"
  b. To control the source of the data fed to the algorithm, the values associated with the **“data”** key are used.
    i. Setting the value associated with the **“dataset_path”** key to **‘’data/rat”** for example points the algorithm to pre-collected data and runs hyperparameter calibration on this data by default.
    ii. For more details on running hyperparameter calibration, consult: **“EduOptimNeurostim/tutorials/Experiment1.ipynb”.**
      "data": {
      "dataset_path": "data/rat",
      "selected_muscles": null
      }
    iii. Alternatively, configuring the values under the **“data”** key can specify the data will be acquired online through the experimental apparatus with Synapse instead of being drawn from a pre-existing dataset.
      "data": {
      "online": true,
      "online_api": "synapse",
      "dataset_path": null,
      "selected_muscles": null
      }
  c. When the data is acquired online through the experimental apparatus, the parameter space must be specified under the **“input_space”** key. In the offline context, the parameter space is automatically inferred from the provided pre-collected data.
    "input_space": {
    "channel_x": [1, 2, 3, 4, 5, 6, 7, 8],
    "channel_y": [1, 2, 3, 4]
    }
  d. To provide an optional prior to the algorithm, the values associated with the **“prior”** key are used.
    i. Setting the value under the **“path”** key specifies where the algorithm looks for the prior and the **“scale”** key controls if the prior is scaled by a factor before it is provided to the algorithm.
    ii. For more details on running the algorithm with a prior, consult **“EduOptimNeurostim/tutorials/Experiment5.ipynb”.**
      "prior": {
      "path": null,
      "scale": null
      }
  e. To control the acquisition function, the values under the **“acquisition”** key are used.
    i. The UCB acquisition function is selected by setting **“ucb”** as value under the **“name”** key.
    ii. To control the κ used in the UCB, the values under the **“kappa”** key are used.
    iii. The default κ used is set by the value under the **“default”** key.
    iv. The κ values evaluated in a hyperparameter calibration procedure are set in the list under the **“values”** key.
    v. The hyperparameter κ is included in the calibration procedure if the value under the **“find_best”** key is set to **“true”.**
      "acquisition": {
      "name": "ucb",
      "kappa": {
      "default": 3.0,
      "values": [1.0, 1.5, 2.0, 2.3, 2.6, 2.9, 3.2, 3.5, 3.8, 4.1, 5.0, 6.0, 7.0, 8.0, 9.0, 10.0],
      "find_best": true}
      }
  f. To control the optimization algorithm, the values under the **“optimization”** key are used.
    i. Specifically, the algorithm used is selected by setting the value under the **“name”** key. **“name”** should remain “**gpbo”** unless the user is interested in alternative algorithms, which the primary publication^1^ demonstrated to be inferior.
    ii. For more details on alternative optimization algorithms, consult **“EduOptimNeurostim/tutorials/Experiment1.ipynb”.**
    iii. Moreover, the number of queries to perform during the algorithmic execution is controlled by the value under the **“max_queries”** key**.**
    iv. The number of times the algorithmic performance is evaluated with a given hyperparameter value in the calibration procedure is controlled by the value under the **“n_repetitions”** key.
    v. The number of queries performed randomly at the beginning of the algorithmic execution is controlled by the value under the **“n_random_steps”** key.
    vi. As with κ, if the value under the **“find_best”** key is set to **“true”**, the number of random queries set under the **“values”** key are evaluated in a hyperparameter calibration procedure.
      "optimization": {
      "name": "gpbo",
      "max_queries": 32,
      "n_repetitions": 30,
      "n_random_steps": {
      "default": 1,
      "values": [1, 2, 3, 4, 5, 7, 10, 15, 20, 25, 30, 32],
      "find_best": false}
      …}
59. In the current setting, you have access to data from previous instances of the same experimental procedure. Use this data to run hyperparameter calibration and determine which values of hyperparameters are optimal.
  a. Make antecedent data available. As indicated in the **“README”**, Windows requires the use of GitBash to run the following command.
    > bash /scripts/download_nhp_rat_dataset.sh data
  b. Adequately populate the **“/config.json”** file.
    i. Point the algorithm to the antecedent data.
      "data": {
      "dataset_path": "data/rat",
      "selected_muscles": null
      }
    ii. Since the hyperparameter κ is particularly consequential for the algorithmic performance, include it in the hyperparameter calibration by setting **“find_best”** to **“true”** under the **“kappa”** key.
      "acquisition": {
      "name": "ucb",
      "kappa": {
      "default": 3.0,
      "values": [1.0, 1.5, 2.0, 2.3, 2.6, 2.9, 3.2, 3.5, 3.8, 4.1, 5.0, 6.0, 7.0, 8.0, 9.0, 10.0],
      "find_best": true}
      }
    iii. To include other hyperparameters in the calibration procedure, simply set their respective **“find_best”** key to **“true”** in the **“/config.json”** file. As specified above, when searching for the best stimulation location, we found the calibration of these other hyperparameters to have minimal impact.
    iv. Change the output path to an appropriate name.
      "output_path": "output/hyperparameter_cali_kappa"
    v. Configure the number of queries by setting **“max_queries”** to 32, resulting in as many queries as there are electrodes in the MEA. This small number of queries has been shown to perform well (see Fig. 2D & Fig. S4C of the primary publication^1^).
      "optimization": {
      "name": "gpbo",
      "max_queries": 32,
      "n_repetitions": 30,
      "n_random_steps": {
      "default": 1,
      "values": [1, 2, 3, 4, 5, 7, 10, 15, 20, 25, 30, 32],
      "find_best": false}
      …}
60. Run hyperparameter calibration. This may take a while.

> python main.py -c config/config.json

61. From the output graph in **“/output/hyperparameter_cali_kappa”**, determine which value of hyperparameter κ leads to highest average performance and populate this value in the **“default”** field under the **“kappa”** key in the **“/config.json”** file.

### Run online optimization procedure | optimizing the ICMS electrode location to maximizes peak EMG amplitude

**Timing: 1 h**

The purpose of this step is to run the algorithm in the implanted rat to optimize the stimulation electrode location used to evoke a maximal peak EMG amplitude in the Tibialis Anterior muscle.

62. Prepare the config file for the online optimization procedure.
  a. In this context, it is important to understand the distinction between *channel* number and the Cartesian coordinates of the electrode location.
    i. In Synapse, stimulation electrodes are addressed as *channels* from 1 to 32. This specifies which of the IZ2 pins outputs the stimulation.
    ii. In contrast, electrodes in the MEA are arranged in a 2D grid indexed as Cartesian coordinates (e.g., (1,1), (1,2) …).
    iii. The mapping from *channel* number to the Cartesian coordinates of the electrode contact depends on the specific headstage used.
    iv. The algorithm represents electrodes in a 2D grid and selects queries at Cartesian coordinates. To provide Synapse with the appropriate *channel* number to stimulate, the mapping must be provided in the form of a **“/config/ch2xy_online.json”** file. The path to this file has to be set in **“/config.json”.**
      "eletrode_mapping_path":"config/ch2xy_online.json"
  b. Specify the input space by listing the possible x and y Cartesian coordinates for the stimulation *channels.*
    "input_space": {
    "channel_x": [1, 2, 3, 4, 5, 6, 7, 8],
    "channel_y": [1, 2, 3, 4]
    }
  c. At this stage, additional parameters, such as the stimulation frequency, can be introduced for online optimization alongside the stimulation location (i.e., electrode contact).
    i. Note that to guarantee the validity of the hyperparameter selection, it is advisable to include these additional parameters in the hyperparameter calibration dataset.
    ii. To achieve this, a semi-synthetic dataset can be constructed by merging the data on stimulation location from the provided "data/rat" dataset with information about the effect of frequency derived from Figure 5 in Watson et al., 2016.^10^
    iii. Specifically, the simplifying assumption that the impact of frequency on the response amplitude is applied as a normalized modulation factor can be made. For instance, for frequencies [100, 200, 300, 400, 500], the modulation factors [0.52, 0.94, 1, 1.05, 1.11] can be derived.
    iv. To include the frequency as a parameter in the dataset construction, the efficacy of the electrode is simply multiplied with this factor. For example, if a response to electrode 13 is 0.4, then this response which was obtained at 300 Hz in the provided dataset is extrapolated to be 0.4 multiplied by 0.52 at 100 Hz. This is repeated for every response in the dataset for every frequency.
      "input_space": {
      "channel_x": [1, 2, 3, 4, 5, 6, 7, 8],
      "channel_y": [1, 2, 3, 4],
      "frequency": [100, 200, 300, 400, 500]
      }
  d. Change the output path to an appropriate name.
    "output_path": "output/online_rat_mapping_2D"
  e. Change the data source.
    "data": {
    "online": true,
    "online_api": "synapse",
    "dataset_path": null,
    "selected_muscles": null
    }
  f. Verify the number of queries that will be effectuated is adequate. Under the **“optimization”** key, **“max_queries”** should still be set to 32.
    "max_queries": 32
  g. If you want to use a prior on the stimulation location efficacy, it can be fed by adding the following value under the **“path”** key.
    i. The **“scale”** key corresponds to a scaling factor for the prior which controls the strength of its influence.
    ii. We have found that in this context, using a scale of 0.5 works best.
      "prior": {
      "path": "priors/rat_mapping_2D.mat",
      "scale": 0.5
      }
63. The following command calls the algorithm online. Press **“enter”** to execute queries.

> python main.py -c config/config.json

64. Before running the algorithm with the animal, dry run the procedure.

**CRITICAL:** Does the hardware work as expected? Does the software work as expected? To test both the above components, connect both stimulation and recording headstages to test wires and immerse the wires in saline. Run the above command. Verify the presence of a stimulation artefact in the recordings.

65. Bring the animal to the space for electrophysiologic experimentation.
66. Connect the stimulation and EMG headstages to the connectors on the head of the animal.
67. Run the above command and maintain the animal in a position where the hindlimb is freely suspended during the 32 queries (a few minutes).

**CRITICAL:** Avoid executing queries when the animal is moving. During the algorithm procedure, observe the evolution of the evoked muscles responses. Different issues can arise here, see **Troubleshooting problem 3–6** below.

68. Return the animal to the cage.
69. Assess the evolution of the exploration and exploitation metrics across queries and the combination of parameter values found as optimal after the last query in the output plots saved in **“/output/online_rat_mapping_2D”.**

## Expected outcomes

Using the method detailed in this protocol, the ICMS electrode location used in the rat motor cortex to evoke maximal EMG activity in the Tibialis Anterior can be effectively optimized. In this setting, because of the mosaic nature of movement representation in the motor cortex,^15^^,^^16^^,^^17^^,^^18^^,^^19^ the parameters-response relationship is quite unsmooth, making optimization harder. However, the use of a 32 electrode MEA restricts the total number of combinations of parameter values in the input space, making optimization easier. Going over the specifics of the current setting, the robustness of the algorithm across datasets with different smoothness of the parameters-response relationship and high-dimensional input spaces has been demonstrated.^1^ Moreover, the method was previously used to optimize diverse stimulation parameters such as the stimulation timing, duration, frequency, pulse width and the spinal site of stimulation (see Fig. 7 & Fig. 8 of the primary publication^1^). Stimulation parameters can also be optimized using other evoked response features, for example the kinematic step height during locomotion (see Fig. 6 of the primary publication^1^). Because of this flexibility, only minor changes are required for the successful adaptation of our method to other neuroprosthetic paradigms.

## Quantification and statistical analysis

### EMG processing

During the online optimization procedure, the EMG signals are recorded at 6 KHz, high-pass filtered at 70 Hz, rectified and low-pass filtered at 30 Hz to obtain the envelope of the signal. Then, 50 ms after the onset of the stimulation (which lasts 43 ms), the peak amplitude of the envelope is extracted. This delay guarantees that a potential stimulation artefact is excluded from the response quantification. This is how the evoked response feature value is obtained.

### Performance plots

As discussed above, the model is evaluated using two metrics computed after each query: the exploration performance and the exploitation performance. The metrics are computed differently in the presence of *ground truth* data such as when the algorithmic procedure is simulated with pre-collected data or in the absence of *ground truth* data such as when the algorithm is used online.

In the presence of *ground truth* data, the exploration metric reports the efficacy of the parameters (e.g., given stimulation electrode) considered optimal by the algorithm in proportion to the optimum. For example, if the exploration performance of the algorithm is 80% after 10 queries, it means that the combination of parameter values currently considered best elicits a response that is 80% of the highest response achieved across all combinations. In the absence of *ground truth* data, the optimum is not known. In this case, instead of being compared to the optimum, the efficacy of the parameters considered optimal by the algorithm is simply reported. ‘‘Exploitation’’ is the capability of targeting effective regions of the input space early and persistently, a likely indication for an effective therapy. In the presence of *ground truth* data, it follows that the exploitation metric reports the efficacy of the current choice of parameters in proportion to the optimum. For instance, if the exploitation performance of the algorithm is 80% after 10 queries, it means that the stimulation delivered at query 10 would elicit a response that is 80% of the highest response achieved across all parameters. In absence of *ground truth* data, the efficacy of the queried parameters is compared to the efficacy of the parameters predicted as the optimal by the model. A performant algorithm procedure will be characterized by both metrics curves climbing across queries. However, exploration and exploitation exist in a trade-off which is controlled by κ. As discussed above, the UCB acquisition function computes a weighted sum of the predicted mean and uncertainty for each combination of parameter values. The hyperparameter κ acts as a weighting factor for the uncertainty. At a stage in the algorithmic procedure, a dataset containing the queries performed so far is provided to the GP-BO and informs the predictions of mean and uncertainty. A given combination (e.g., simulation electrode number 16) is selected to be queried next when its associated sum is higher as compared to the sum associated with other combinations. Using a small value of κ will make the sum dominated by the predicted mean. The combination will be queried if it has a higher predicted mean, favoring exploitation. Using a large value of κ will make the sum dominated by the uncertainty. The combination will be queried if it still has higher uncertainty despite the data available, favoring exploration. While a degree of exploration is necessary to discover optimal combinations, an excessive bias toward uncertainty reduction can detrimentally affect exploitation performance due to more frequent tests in suboptimal regions of the parameter space (see Fig. S1 in the primary publication^1^).

When running a hyperparameter calibration procedure, to compare the overall performance when using specific values of hyperparameter, the performance at the final query is used to encapsulate the whole curve. The final query performances are averaged over replicates and plotted for each hyperparameter value.

## Limitations

While the GP-BO algorithm is an effective optimization technique, it is not guaranteed that it will find the global optimum in a viable number of queries.

Moreover, since the algorithm only maximizes the evoked response feature, it may lead to unforeseen or undesired effects. For example, by searching the electrode location to evoke maximal EMG activity in a muscle of interest, the electrode predicted as optimal might lead to the coactivation of the muscle of interest and another *off-target* muscle. If this is problematic, another evoked response feature which better recapitulates the desired features of response might be considered (e.g., step height).

Additionally, stochasticity intervenes at different levels in the optimization procedure. The search procedure starts with a given number of random queries. Also, the EMG responses display some level of noise. However, the performance of the algorithm reported in the primary publication^1^ are averages obtained over replicates. It follows that it may be necessary to repeat the optimization procedure more than once to gain a sufficient level of confidence in its recommendation of optimal parameter values.

## Troubleshooting

### Problem 1

During the surgery, before the array implantation, the brain of the rat brain shows signs of edema (related to Step 35 in Rat Implantation Surgery).

### Potential solution

• Consider opening cisterna magna with a small incision to relieve the intracranial pressure.
• Consider adding a mannitol injection to your surgical procedure.

### Problem 2

Problems in configuring the Synapse software (related to Step 54 in experimental apparatus).

### Potential solution

• Consult the documentation at https://www.tdt.com/files/manuals/SynapseManual.pdf.

### Problem 3

During the algorithmic execution, the stimulation seems ineffective (related to Step 67 in run online optimization procedure).

### Potential solution

• Check the hardware and verify the stimulator is appropriately armed.
• Try the stimulation with a set of parameters that are known to produce a potentially suboptimal though visible response. This step helps to rule out hardware problems or failure of the implants.
• When the parameter space is large and most combinations of parameter values are ineffective, it is possible that most stimulation effectuated by the algorithm will be ineffective. In this case the number of queries effectuated is not sufficient for the total number of combinations of parameter values of the problem.

### Problem 4

The algorithm does not seem to converge on a combination of parameter values within the allowed number of queries (related to Step 67 in run online optimization procedure).

### Potential solution

• Consider effectuating a larger number of queries.
• Consider reducing the size of the parameter space.
• In the case where the two options above are not possible, try reducing the value of k, and evaluate if even given the small number of queries the algorithm is still performant (i.e., there is an increase in stimulation efficacy over queries). If not, the problem might not be solved with the limited number of queries.

### Problem 5

The algorithm converges too early and/or on a clearly suboptimal combination of parameter values (related to Step 67 in Run Online Optimization Procedure).

### Potential solution

• Raise the value of κ.

### Problem 6

The evoked responses are contaminated by the spontaneous movements of the animal (related to Step 67 in run online optimization procedure).

### Potential solution

• Although the algorithm is robust to occasional spurious responses, the optimization works better if the parameters-response relationship remains stable. Since stimulations are manually triggered, make sure to press the triggers while the rat is at rest. Try placing the rat in a more relaxing position. To facilitate this, it is best to habituate the rat to human contact before the experiments.

## Resource availability

### Lead contact

Further information and requests for resources should be directed to and will be fulfilled by the lead contact, Numa Dancause (numa.dancause@umontreal.ca).

### Technical contact

Léo Choinière (leo.choiniere@umontreal.ca)

### Materials availability

This study did not generate new unique reagents.

### Data and code availability

• The complete cortical mapping dataset consisting of 4 NHPs and 6 rats is publicly available as of the date of publication at the following Open Science Framework Repository Database: https://osf.io/54vhx/
• All original code has been deposited at GitHub and is publicly available with a variety of tutorials. See: https://doi.org/10.5281/zenodo.10494808 or https://github.com/mbonizzato/EduOptimNeurostim for a Python-based library and educational material (Jupyter Notebook tutorials) or https://github.com/mbonizzato/OptimizeNeurostim/ for MATLAB-based implementations.
• Any additional information required to reanalyze the data reported in this paper is available from the lead contact upon request.
